# Free Energy Calculations using a Swarm-Enhanced Sampling Molecular Dynamics Approach

**DOI:** 10.1002/cphc.201500524

**Published:** 2015-09-29

**Authors:** Kepa K Burusco, Neil J Bruce, Irfan Alibay, Richard A Bryce

**Affiliations:** [a] Manchester Pharmacy School, University of ManchesterOxford Road, Manchester, M13 9PT, UK; [b] Heidelberg Institute for Theoretical Studies (HITS gGmbH)Schloss-Wolfsbrunnenweg 35, 69118, Heidelberg, Germany

**Keywords:** enhanced sampling, free energy calculations, kinetic substates, molecular dynamics, swarm

## Abstract

Free energy simulations are an established computational tool in modelling chemical change in the condensed phase. However, sampling of kinetically distinct substates remains a challenge to these approaches. As a route to addressing this, we link the methods of thermodynamic integration (TI) and swarm-enhanced sampling molecular dynamics (sesMD), where simulation replicas interact cooperatively to aid transitions over energy barriers. We illustrate the approach by using alchemical alkane transformations in solution, comparing them with the multiple independent trajectory TI (IT-TI) method. Free energy changes for transitions computed by using IT-TI grew increasingly inaccurate as the intramolecular barrier was heightened. By contrast, swarm-enhanced sampling TI (sesTI) calculations showed clear improvements in sampling efficiency, leading to more accurate computed free energy differences, even in the case of the highest barrier height. The sesTI approach, therefore, has potential in addressing chemical change in systems where conformations exist in slow exchange.

##  Introduction

Free energy simulation methods such as free energy perturbation (FEP) and thermodynamic integration (TI) are increasingly important tools in the pursuit of molecular design.^1^,^2^ Despite recent advances, however, the computation of free energies remains challenging for many systems, where the free energy change may depend on multiple distinct substates separated by non-negligible energy barriers.^3^ Suitably sampling kinetically distinct but thermodynamically relevant substates is challenging, particularly where prior knowledge of their existence is lacking.^4^ Considerable effort has been invested in tackling the problem of pseudo-ergodicity in simulation-based free energy calculations.^3^,^5^–^7^ A range of sampling methodologies have been proposed; for example, approaches based on TI include the independent trajectory TI (IT-TI) method,^8^ enhancing sampling by coupling to accelerated molecular dynamics (aMD)^9^ or replica-exchange-based methods such as RETI.^10^ Recently, we have explored a multi-copy molecular dynamics method designed to improve conformational exploration of rugged energy landscapes.^11^ A development out of the SWARM-MD approach of Huber and van Gunsteren,^12^,^13^ we term this method swarm-enhanced sampling molecular dynamics (sesMD).^11^ The sesMD method links multiple simulation replicas into a swarm, using attractive and repulsive pair potentials acting on dihedral angles to promote barrier crossing into alternative energy minima. Application of sesMD has led to enhanced sampling of the conformations of small-molecule systems and a protein kinase,^11^ as swarm replicas cooperatively sample a greater volume of phase space by steering each other across potential energy barriers.

The possibility of harnessing the conformational exploration afforded by swarm-coupled trajectories to improve the accuracy of free energy calculations has been recognised previously.^14^ Here, we assess the predictive quality of free energy estimates by combining the sesMD approach with a dual topology TI framework. We illustrate the approach of this method, denoted hereafter as sesTI, for the diagnostic case of the butane-to-butane alchemical transition in water. Alkane transformations of this type can suffer from errors in the calculated free energy change, owing to inadequate sampling of the hydrocarbon’s internal rotations.^9^,^15^–^17^ For the transition, we study three butane potentials of growing energy barrier height between rotamers to represent increasingly distinct energetically low-lying substates. The sesTI scheme is compared with the TI and IT-TI methods, directly examining the effect of swarm-coupling replicas on their sampling of kinetically separated substates.

##  Theory

According to the TI approach, the Helmholtz free energy change Δ*A* of a transition along a coordinate *λ* is obtained by using Equation 1:

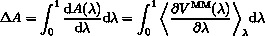
1

where *λ*=0 and *λ*=1 represent the initial and final states of the transition, respectively, and *V*^MM^ is the molecular mechanical potential energy of the system. Thus, the total difference in free energy Δ*A* can be obtained by using an appropriate quadrature scheme to integrate over *λ* from *λ*=0 to *λ*=1 the ensemble averaged 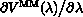
values. For the sesTI method, we now consider the computation of Δ*A* according to the TI approach, but within a sesMD framework, that is, for a swarm of *M* simulation replicas *α*. We firstly define the total potential, *V* ^tot^, acting on this swarm within a sesMD simulation, as shown in Equation 2:

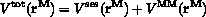
2

where vector **r^M^** is the 3*NM* dimension vector describing the coordinates of *N* atoms in *M* replicas. Here, *V* ^MM^(**r^M^**) is the sum of force field potentials, 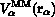. The swarm-enhanced sampling (ses) potential, *V* ^ses^, is defined by Equation 3:

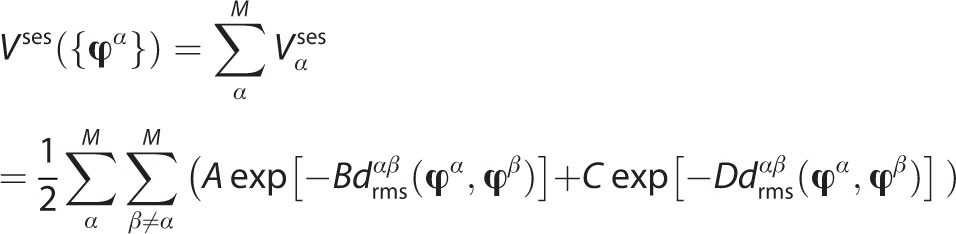
3

where A–D are suitably calibrated parameters for attractive (A, B) and repulsive (C, D) terms and 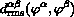
is the root-mean-square dihedral angle distance of *K* dihedrals *j* between swarm members *α* and *β*, namely 
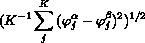
.

If we consider the contribution of replica *α*, 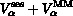, an expectation value of dA/dλ for the mutation of a single butane molecule can be obtained from the sesMD ensemble average of 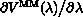
at *λ* by applying the approach of Torrie and Valleau^18^ to recover a Boltzmann weighted average according to Equation 4:

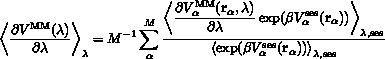
4

Here, we assume that the swarm replicas are weakly coupled, such that their contributions are re-weighted according to 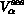. A further approximation is possible if one assumes that, given sufficient sampling, the time average over a given individual replica will converge to the replica average. Applying this assumption to the context of our sesMD system leads to the expression in Equation 5:

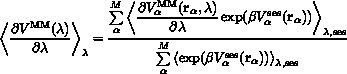
5

In this work, we compare the performance of sesTI with TI and with the non-interacting multi-replica IT-TI approach.^8^

## Computational Details

MD and TI simulations were performed by using the *sander* module of the AMBER12 software package;^19^ sesMD and sesTI calculations were conducted by using a modified version of the *sander* module of AMBER11.^20^ Three model sets of butane parameters were used, each with zero partial charges on the butane atoms. In the first, **b1**, the bonded and van der Waals parameters were taken from the GAFF force field.^21^ In systems **b2** and **b3**, the torsional barrier heights were increased from their GAFF values (Table S1, Figure S1); butane was solvated in a rectangular box of 584 TIP3P water molecules.^22^ Periodic boundary conditions were employed, with a particle-mesh Ewald (PME) treatment^23^,^24^ and a 9 Å cut-off for short-range non-bonded interactions. All MD calculations used a 1 fs time step and the SHAKE algorithm^25^ was applied to constrain solvent bonds. The temperature and pressure were controlled by using Langevin dynamics,^26^ with a collision frequency of 2 ps^−1^ and a Berendsen barostat^27^ with a coupling constant of 2 ps.

**Figure 1:**
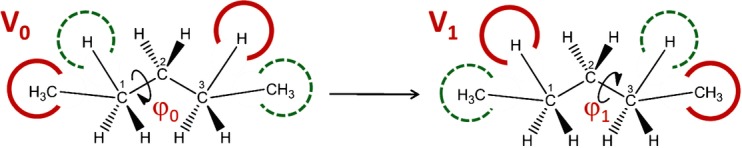
Figure Butane-to-butane alchemical transformation through the dual topology approach. Active groups (in red) and inactive groups (in green) coexist and alter as mixing parameter *λ* evolves from state 0 to 1. Corresponding central C-C-C-C dihedrals *φ*_0,_ and *φ*_1_ for states 0 and 1 are also shown.

Free energy calculations for the butane-to-butane transition for **b1**, **b2** and **b3** employed the dual topology approach^28^ and soft-core potentials.^29^ For TI, IT-TI and sesTI, a straightforward linear scaling^30^ of the mutating groups was used, employing a *λ* path of 13 points (*λ*=0.01, 0.05, 0.10, 0.20, 0.30, 0.40, 0.50, 0.60, 0.70, 0.80, 0.90, 0.95 and 0.99). Each window was equilibrated for 800 ps by using rounds of NPT and NVT dynamics. Subsequently, the final geometry from this trajectory was replicated 12 times, with initial velocities assigned from a 298 K Maxwell–Boltzmann distribution and equilibrated for 100 ps. For IT-TI calculations, this was followed by 5 ns NVT production dynamics at 298 K. For sesTI calculations, a ses potential was applied to the central *φ*_0_ and *φ*_1_ angles of butane (Figure 1). To explore the differing energy landscapes of systems **b1**, **b2** and **b3**, we applied distinct sets of (A, B) and (C, D) parameters (Table 1). The ses potentials, with a repulsive and longer-range attractive profile were empirically fitted to ensure a satisfactory frequency of crossing over the *gauche*–*trans* energy barrier for each system. Reflecting the greater barrier height from **b1** through to **b3**, the magnitude of pre-exponential parameters A and C correspondingly increased (Table 1). Prior to a 5 ns production simulation of the 12-replica swarm of each *λ*, a further 200 ps equilibration was applied, where the ses potential was gradually increased from zero to its full strength (Table 1). Coordinates were saved for analysis every 1.0 ps and energies every 0.1 ps.

**Table 1:** Set of ses potential parameters used in sesTI calculations for systems b1, b2 and b3.

Model	*A* [kcal mol^−1^]	*B* [rad^−1^]	*C* [kcal mol^−1^]	*D* [rad^−1^]
**b1**	−10.0	0.2	15.0	1.0
**b2**	−50.0	0.2	50.0	0.8
**b3**	−100.0	1.0	200.0	1.5

Free energy estimates were computed by using the approach by Steinbrecher et al.^30^,^31^ to obtain independent samples from the windowing trajectories, based on the autocorrelation time *τ* of d*V/*dλ. The standard error of the mean, *σ*_SEM_, was computed as 
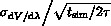
, where *σ*_d*V*/d*λ*_ is the standard deviation and *t*_sim_ is the total length of the simulation. For IT-TI, free energy estimates were obtained by considering an average over the free energies computed from each of the 12 replicate simulations. For sesTI, free energies were computed from Equations (4) or (5) above. For the latter, bootstrap sampling^32^,^33^ from the combined 12 trajectories was applied, using uniform sampling and the Mersenne Twister pseudo-random-number generator mt19937.^34^ 1.2×10^5^ configurations were obtained for each *λ*. Geometrical analysis of trajectories used *cpptraj*^35^ from the AMBER suite.

##  Results

We consider the ability of sesTI to accurately calculate the free energy change for the transformation of *n*-butane to *n*-butane in explicit aqueous solvent (Figure 1). The path for this test case alchemical transformation involves mutation of the methyl group on carbon C_1_ of butane to a hydrogen atom, and vice versa for C_3_ (Figure 1). However, regardless of the path *λ* and force field employed, the free energy difference between these states should be zero. We employ here a dual topology approach to TI; thus, there is the coexistence of mutating groups in the initial reference butane state *λ*=0 with potential *V*_0_, and final target butane state *λ*=1 with potential *V*_1_, albeit without interacting during the MD simulation. The remaining atoms, common to both states, evolve according to the potential 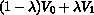. Due attention is given to the alchemical end points by using soft-core van der Waals potentials for butane and the division of *λ* into 13 suitably spaced windows.

Nevertheless, the accuracy of the TI calculation also relies on the adequate sampling of conformations relevant to the reference and target states across the whole *λ*-dependent path, in particular around C-C-C-C torsions *φ*_0_ for *λ*=0 and *φ*_1_ for *λ*=1 (Figure 1). These torsion angles can potentially adopt *trans* (*t*) and *gauche* (*g*^*+*^ and *g*^−^) rotamers. In this study, we consider three different butane potentials, **b1**, **b2** and **b3**. For all three systems, the *t* well lies 1 kcal mol^−1^ lower in energy than the *g*^*+*^ and g^−^ minima (Figure S1). However, the total potential energy barriers from the *t* to *g*^*+*^/*g*^−^ wells (i.e. including torsional and non-bonded terms) are progressively enlarged from approximately 4 kcal mol^−1^ (**b1**) to 6 kcal mol^−1^ (**b2**) and 8 kcal mol^−1^ (**b3**), as shown by their rotational profiles (Figure S1). To accentuate the issue of sampling, we initially assign *t* and *g*^−^ conformations to butane torsions *φ*_0_ and *φ*_1_, respectively. Consequently, these systems present increasingly challenging access to thermodynamically relevant states for the butane-to-butane mutation. For the transformations of these three systems, we compare the performance of sesTI with 1) TI calculations based on Δ*A* estimates from the 12 individual MD trajectories and 2) Δ*A* from averaging over these 12 TI simulations, namely the IT-TI approach.^8^

**Figure 2:**
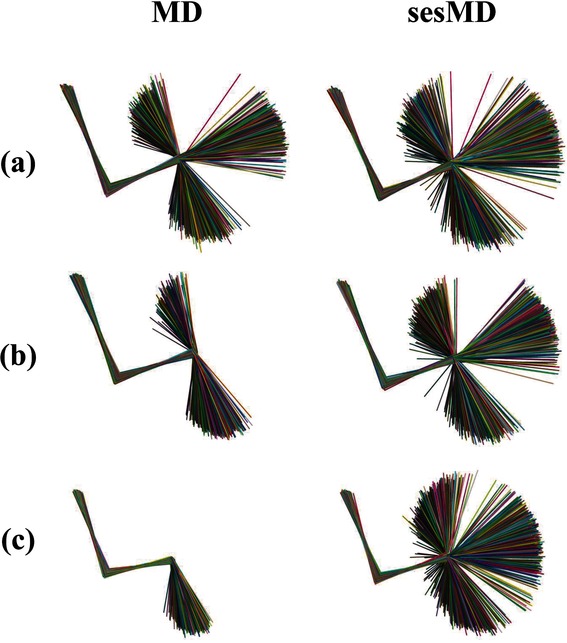
Figure Sampling of dihedral angle *φ*_0_ at *λ*=0.01 in butane systems a) b1, b) b2 and c) b3 from 5 ns of 12 independent trajectories for IT-TI (MD) and 12-replica swarm trajectories (sesMD); snapshots at 50 ps intervals and individually coloured for clarity.

As an initial indication of the ability of unbiased MD and sesMD to surmount energy barriers in **b1**, **b2** and **b3**, we consider the 5 ns of simulation at *λ*=0.01, that is, the λ point closest to the reference state. Superposition of equal-spaced snapshots from the 12 unbiased MD trajectories of IT-TI at *λ*=0.01 indicates that, for **b1**, all three *φ*_0_ rotamers of butane are explored (Figure 2 a); for **b2**, only *t* and *g*^−^ (Figure 2 b) are explored and only the initial conformation *t* for **b3** (Figure 2 c). By comparison, the *λ*=0.01 window simulation, using 12-replica sesMD samples shows all three rotamers for all three butane models (Figure 2 a–c). The three models apply ses potentials of increasing strength from **b1** to **b3** (Table 1); it appears that the broadest coverage of *φ*_0_ space is found for **b3** (Figure 2 c).

From this preliminary assessment of underlying sampling, we now turn to consider the estimates of the free energy change for the butane-to-butane transformation furnished by TI, IT-TI and sesTI approaches. We label the 12 independent TI calculations as TI-01 to TI-12 (Table 2). For the butane system with the lowest energy barriers, **b1**, we find that all 12 TI calculations provide a predicted Δ*A* close to zero, with a range of −0.06 to 0.03 kcal mol^−1^ (Table 2). Standard errors are uniformly 0.01 kcal mol^−1^ in value. Correspondingly, the combined IT-TI estimate from these individual TI calculations is −0.01±0.01 kcal mol^−1^ (Table 2), again in good agreement with the theoretical value of zero. For the application of sesTI in enhancing the backbone torsions of butane during the alchemical change, the estimated Δ*A* is 0.01±0.01 kcal mol^−1^ when re-weighting according to Equation (4), and 0.02±0.01 kcal mol^−1^ when using Equation (5). Thus, the estimates of both schemes are in agreement with each other and close to zero.

**Table 2:** Free energy differences Δ*A* and standard errors of alchemical transformation for systems b1, b2 and b3 by using TI, IT-TI and sesTI methods.

Method	Δ*A* [kcal mol^−1^]		
	b1	b2	b3
TI-01	−0.04±0.01	0.24±0.01	0.69±0.01
TI-02	−0.04±0.01	0.19±0.01	0.70±0.01
TI-03	0.03±0.01	0.27±0.01	0.67±0.01
TI-04	0.01±0.01	0.40±0.01	0.70±0.01
TI-05	0.00±0.01	0.27±0.01	0.68±0.01
TI-06	0.02±0.01	0.26±0.01	0.71±0.01
TI-07	−0.06±0.01	0.14±0.01	0.71±0.01
TI-08	−0.02±0.01	0.11±0.01	0.69±0.01
TI-09	−0.04±0.01	0.11±0.01	0.70±0.01
TI-10	0.01±0.01	0.24±0.01	0.72±0.01
TI-11	0.03±0.01	0.18±0.01	0.70±0.01
TI-12	0.00±0.01	0.16±0.01	0.69±0.01
IT-TI	−0.01±0.01	0.21±0.02	0.70±0.00
sesTI/Eq. (4)	0.01±0.01	−0.03±0.03	−0.03±0.09
sesTI/Eq. (5)	0.02±0.01	0.05±0.03	0.11±0.23

**Figure 3:**
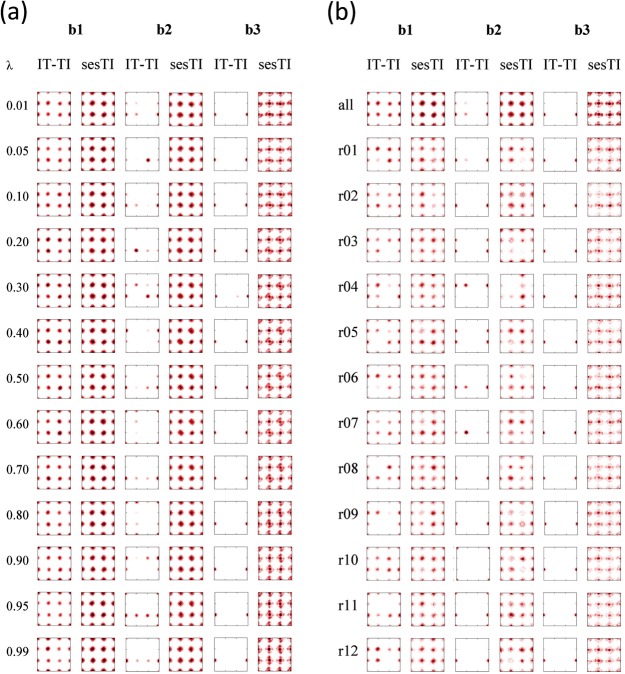
Figure Population of *φ*_0_*φ*_1_ rotamers of butane during IT-TI or sesTI transformation in b1, b2 and b3 systems a) as a function of *λ* (combined replicas) and b) as a function of replicas r01 to r12 (for *λ*= 0.01) and their sum (“all”). Abscissa is *φ*_0_ and ordinate is *φ*_1_. Both axes range from −180° to 180°.

**Figure 4:**
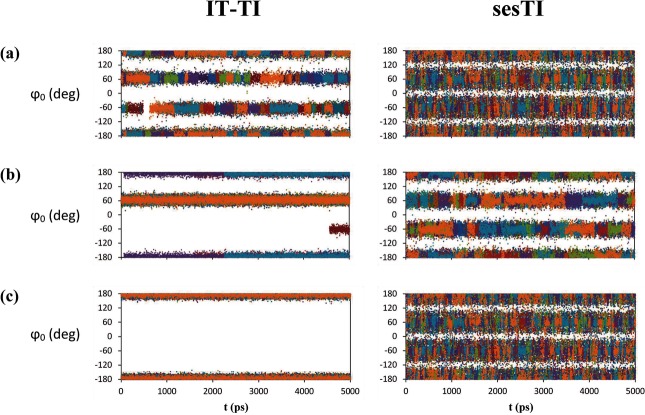
Figure Time series of dihedral angle *φ*_0_ at *λ*=0.05 for butane systems a) b1, b) b2 and c) b3 systems for IT-TI and sesTI alchemical transformations. Windows include all replica contributions.

**Figure 5:**
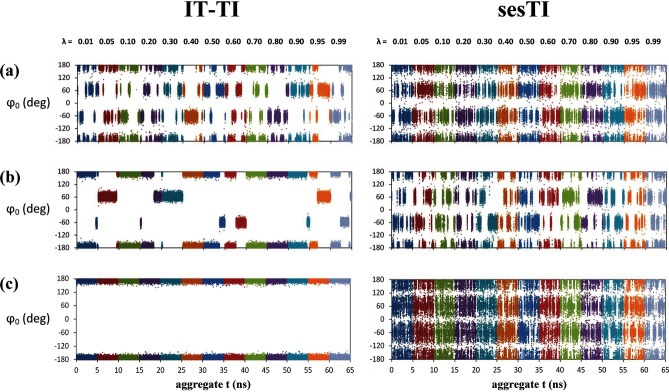
Figure Time series of dihedral angle *φ*_0_ of a) b1, b) b2 and c) b3 systems across *λ* for a single replica of IT-TI (TI-01) and a single replica of sesTI (r01) alchemical transformation.

**Figure 6:**
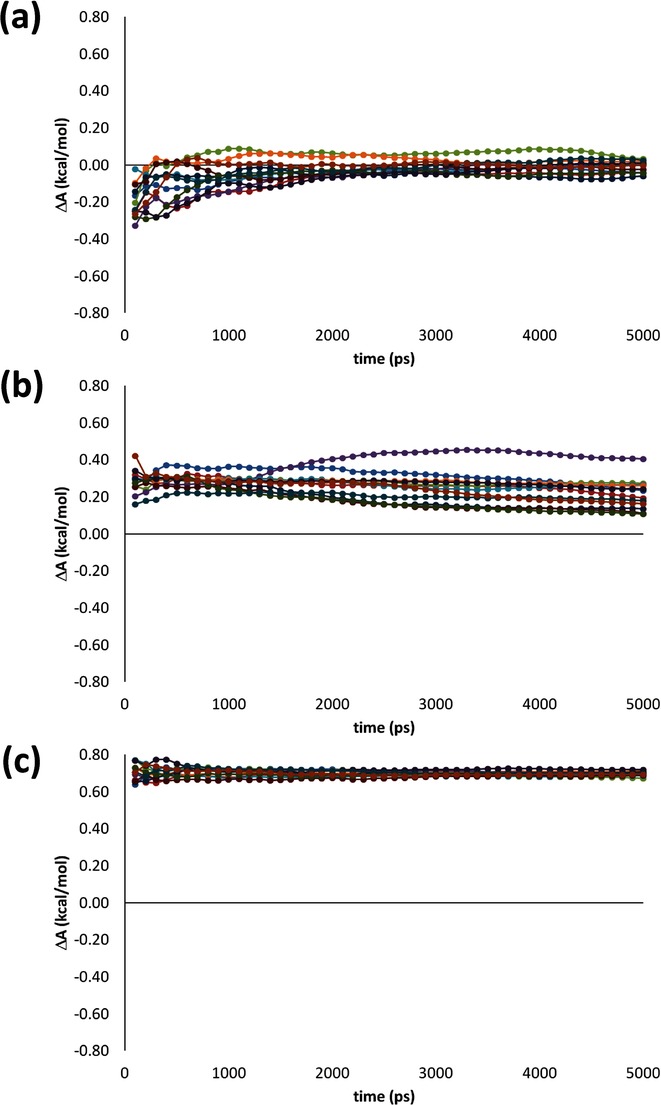
Figure Convergence of free energy difference estimates as a function of simulation length of 12 independent replica TI calculations for systems a) b1, b) b2 and c) b3.

**Figure 7:**
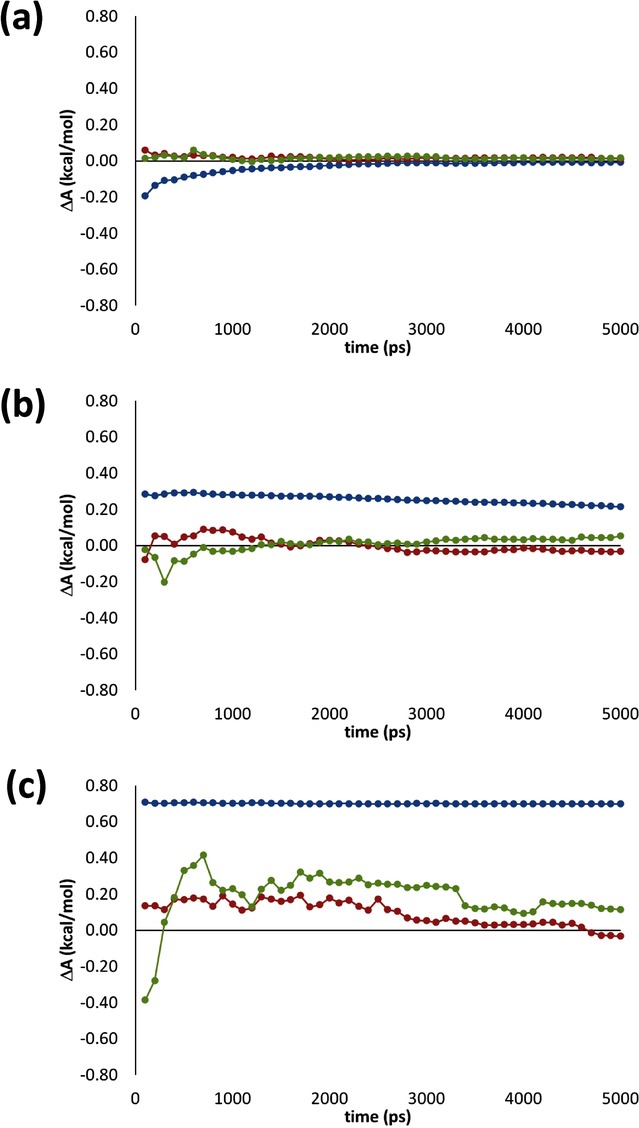
Figure Convergence of free energy difference estimates as a function of simulation length for butane-to-butane transitions of a) b1, b) b2 and c) b3 systems from IT-TI (blue) and sesTI by using Equation (4) (red) and Equation (5) (green).

Reflecting the rotamer sampling at *λ*=0.01, considered earlier, the underlying sampling of *φ*_0_ and *φ*_1_ for both IT-TI and sesTI simulations appear to explore *t*, *g*^*+*^ and *g*^−^ minima for **b1** across all *λ* (first two columns of Figure 3 a) and with each replica (first two columns of Figure 3 b), although to a greater extent for sesTI. It also appears that the transitions between wells are frequent in the 12 TI simulations. This is exemplified by exploration of the swarm in the *λ*=0.05 window (Figure 4 a) and sampling across the *λ* range (Figure 5 a); for IT-TI, an average transition frequency of 2.8 ns^−1^ in *φ*_0_ are found for the *λ* window simulations (Table 3). For the swarm-coupled sesTI simulations, this frequency increases five-fold to 15.5 ns^−1^ (Table 3 and Figure 4 a). This difference in sampling frequency between IT-TI and sesTI reflects the rate at which Δ*A* estimates converge as a function of simulation time. The individual TI estimates of Δ*A* converge to comparable values that are within 0.2 kcal mol^−1^ of each other, at around 2.5 ns of MD sampling for each *λ* window (Figure 6 a). This reduces further to within 0.15 kcal mol^−1^ at 5 ns. Similarly, the IT-TI average based on these individual Δ*A* estimates converges within 3 ns to −0.01 kcal mol^−1^ (Figure 7 a); the standard error also appears low and stable at 0.01 kcal mol^−1^ from 2 ns (Table 4). Interestingly, both sesTI estimates provide average Δ*A* values of close to zero when using *λ* window simulation lengths of only 100 ps (Figure 7 a); specifically, Δ*A* is 0.06±0.06 kcal mol^−1^ and 0.01±0.06 kcal mol^−1^ for Equation (4) and Equation (5), respectively (Table 4).

**Table 3:** Frequency of transitions of C-C-C-C dihedrals *φ*_0_ and *φ*_1_ in b1, b2 and b3 from IT-TI and sesTI calculations, averaged over replica and *λ*. Standard deviations in parentheses.

Model	Method	Frequency of dihedral transition [ns^−1^]	
		*φ*_0_	*φ*_1_
**b1**	IT-TI	2.8 (0.2)	2.9 (0.2)
	sesTI	15.5 (0.8)	15.4 (0.7)
**b2**	IT-TI	0.2 (0.1)	0.2 (0.1)
	sesTI	4.7 (0.2)	5.0 (0.4)
**b3**	IT-TI	0.0 (0.0)	0.0 (0.0)
	sesTI	35.2 (0.7)	36.2 (1.0)

**Table 4:** Free energy differences Δ*A* and standard errors [kcal mol^−1^] of alchemical transformation for systems b1, b2 and b3 by using TI, IT-TI and sesTI methods.

*t* [ns]	IT-TI	sesTI [Eq. (4)]	sesTI [Eq. (5)]
**Δ*A* for b1**			
0.1	−0.20±0.03	0.06±0.06	0.01±0.06
1.0	−0.05±0.02	0.02±0.02	0.01±0.02
2.0	−0.03±0.01	0.01±0.02	0.02±0.01
3.0	−0.01±0.01	0.02±0.01	0.02±0.01
4.0	−0.01±0.01	0.02±0.01	0.01±0.01
5.0	−0.01±0.01	0.01±0.01	0.02±0.01
**Δ*A* for b2**			
0.1	0.28±0.02	−0.08±0.08	−0.02±0.14
1.0	0.28±0.01	0.07±0.06	−0.03±0.07
2.0	0.27±0.02	0.03±0.04	0.03±0.04
3.0	0.25±0.02	−0.03±0.04	0.02±0.04
4.0	0.23±0.02	−0.02±0.04	0.03±0.03
5.0	0.21±0.02	0.03±0.03	0.05±0.03
**Δ*A* for b3**			
0.1	0.71±0.01	0.14±0.23	−0.39±0.21
1.0	0.70±0.01	0.14±0.11	0.23±0.18
2.0	0.70±0.00	0.18±0.11	0.27±0.25
3.0	0.70±0.00	0.05±0.09	0.25±0.21
4.0	0.70±0.00	0.03±0.08	0.09±0.23
5.0	0.70±0.00	−0.03±0.09	0.11±0.23

In the second butane system, **b2**, the barrier between *t* and *g*^*+*^ is approximately 2 kcal mol^−1^ higher than for **b1**. The 12 individual TI calculations for **b2** provide a more variable prediction of Δ*A*, ranging from 0.11 to 0.40 kcal mol^−1^ (Table 2). Indeed, the latter value is provided by calculation TI-04, which diverges somewhat from other replicas after 2 ns of MD (Figure 6 b). Nevertheless, it is evident that all replicas are only gradually approaching the correct Δ*A* value and require longer than 5 ns of MD per window (Figure 6 b). Reflecting this, the overall IT-TI estimate of Δ*A* is 0.21±0.02 kcal mol^−1^ (Table 2). For sesTI, the computed Δ*A* is closer to zero, calculated as −0.03±0.03 kcal mol^−1^ and 0.05±0.03 kcal mol^−1^ by using Equations (4) and (5), respectively (Table 2). The sesTI estimates of Δ*A* converge more slowly for **b2** compared to **b1**, but appear to stabilise after 1.6 ns of simulation for each *λ* (Figure 7 b). The standard errors in both re-weighting schemes converge by 2 ns (Table 4). The augmented rotational barriers in **b2** lead to decreased sampling between rotamers, that is, transition frequencies drop by over a factor of ten for IT-TI simulations and by a third for sesTI simulations (Table 3 as well as Figures 4 b and 5 b). Consequently, within the 5 ns window, where 12 TI replicas observe only one change of rotamer in **b2** on average, the swarm of sesTI samples 24 such transitions. Clearly, this restricts the overall coverage of the three rotameric states by IT-TI (Figure 3 b and 5 b) and their relative contributions to Δ*A*. This contrasts with the sesTI window, where each simulation samples all three rotamers (Figure 3 b).

Finally, we consider the **b3** model of butane, with a further 2 kcal mol^−1^ increase in *t→g*^−^ barrier height. Interestingly, the Δ*A* estimates from the 12 TI simulations agree closely with one another, with a narrow range of 0.67–0.72 kcal mol^−1^ (Table 2). The corresponding IT-TI estimate is 0.70±0.004 kcal mol^−1^. Furthermore, these individual (Figure 6 c) and average TI estimates of Δ*A* (Figure 7 c) and their standard errors (Table 4) converge very rapidly with the *λ* window simulation length. By contrast, the sesTI estimates remain reasonable approximations to zero, with values of −0.03±0.09 and 0.11±0.23 kcal mol^−1^ when using Equations (4) and (5), respectively (Table 2). As expected, the convergence of these sesTI estimates of Δ*A* and associated standard deviations for **b3** is slower than for **b1** and **b2** (Table 4). Thus, the Δ*A* computed by using Equation (4) may be converged after 3 ns, but Δ*A* from Equation (5) still experiences significant shifts after this time (Figure 7 c). The larger errors associated with the **b3** system (ca. 0.2 kcal mol^−1^; Table 4) appear to stem from the greater variation in 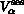
values sampled, with a range in 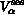
of 16 kcal mol^−1^, as compared to values of 6 and 11 kcal mol^−1^ for **b1** and **b2**, respectively. This arises as a function of the steeper repulsive nature of the ses potential applied to **b3**, such that small changes in the dihedral can lead to larger changes in energy.

The origin of the 0.70±0.004 kcal mol^−1^ difference in free energy for **b3** butane in states 0 and 1 when using IT-TI is immediately apparent from the complete absence of dihedral transitions found in these simulations (Table 3 as well as Figures 3 b, 4 c and 5 c). Consequently, Δ*A* computed from IT-TI corresponds to the difference in stability of a *t* and *g*^−^ conformer of butane. However, under the influence of the cooperative swarm of replica trajectories, the sesTI simulations sample the three rotamers of butane through each replica (Figures 3 b and 4 c) and for each *λ* (Figures 3 a and 5 c). Under the ses potential applied to **b3**, the highest frequency of dihedral transitions for the butane models is found, with a value of 35.2 ns^−1^ for *φ*_0_ (Table 3), permitting the sampling required to obtain estimates of free energy close to zero for the butane-to-butane transformation.

##  Discussion

Free energy calculations constitute valuable tools in modelling chemical processes, for example, in predicting protonation state, solute partitioning between immiscible liquids and molecular association in the condensed phase. Free energy calculations, however, are prone to a number of potential sources of error, principally from the choice of model for the molecular system (e.g. force field, solvent model and treatment of electrostatic interactions) and from finite sampling. By using the butane-to-butane transformation, we focus on the consequences of limited exploration of phase space. Here, errors arise from the omission of conformational regions that are important contributors to the difference in free energy between two states. In the three butane models considered, the *t*, *g*^*+*^ and *g*^−^ energy wells are low lying and contribute thermodynamically to an overall Δ*A* for a butane-to-butane transition of zero. For **b1**, the moderate energy barrier between *t* and *g^+^/g*^−^ rotamers is adequately traversed by unbiased MD simulations of 3 ns for each *λ* window. For the 12 replica IT-TI method, we obtain a Δ*A* estimate of −0.01 kcal mol^−1^ (Table 2). However, for the increasingly kinetically distinct **b2** and **b3** models, the quality of sampling erodes and consequently the free energy estimates deviate to 0.21 and 0.70 kcal mol^−1^, respectively. In the latter case, entire rotamers are omitted.

Accessing these important, but sometimes hidden, substates can be a major issue for free energy methodologies.^4^,^36^ As an approach to reducing this source of error, we evaluated a TI approach based on sesMD for conformational sampling. Multiple MD simulations of butane are coupled through their torsion angles by using a ses potential with attractive and repulsive components [Eq. (3)]. The resultant sesTI calculations of Δ*A* for **b1**, **b2** and **b3** provide good sampling of all three butane rotamers and estimates close to zero (Table 2). More frequent barrier crossing is also observed (Table 3) as swarm replicas transition between wells and stimulate crossings in neighbouring replicas. For **b1**, sesTI convergence appears improved over TI or IT-TI calculations, such that shorter *λ* window simulation times can be employed. For **b2** and **b3**, IT-TI calculations converge more quickly than sesTI, but to an incorrect pseudo-converged value, thus providing precision, that is, a lower statistical error associated with the trapped states, but not accuracy, owing to significant systematic error. Consequently, correction of the IT-TI estimates for **b2** would require the application of much longer unbiased simulations for each *λ* window, which appears beyond reach for the energy landscape of **b3**.

The improved sampling of butane rotamers in states 0 and 1 by using sesTI is evident across *λ* and the replicas (Figure 3). For all 13 *λ* windows, each rotamer within the space of *φ*_0_ and *φ*_1_ are sampled for **b1**, **b2** and **b3** by using sesTI, contrasting with sporadic transitions for **b2** and no transitions for **b3** (Figure 3 a). This good coverage in sampling over *λ* is the result of slightly differing *φ*_0_*φ*_1_ distributions (averaged over *λ*) for each of the sesMD replicas r01 to r12 (Figure 3 b). The resulting aggregate distributions (“all” in Figure 3) show a broader sampling around each rotamer compared to IT-TI, sampling the higher-energy sides of the wells. This would be particularly important for situations where the minima are located at different geometries in states 0 and 1.

For sesTI, we adopted two different re-weighting schemes, according to Equation (4) or Equation (5). In the first scheme, derived from an assumption of weak coupling between replicas, the replicas are re-weighted according to the biasing influence of the ses potential, 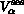. Equation (4) re-weights on a replica basis, such that the dominance of any single replica to the computed average is effectively minimised. In the second scheme, derived from the work of Malevanets and Wodak,^37^ the further assumption is that of ergodicity in the replica trajectories, such that the time average over a replica will converge to the average over replicas. Resultantly, the average computed by using Equation (5) can accentuate the dominance of individual replica contributions to 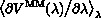. We find that the two weighting schemes are in general agreement with each other, except for the case of the highest energy barrier system, **b3**. Here, it is possible that the strong potential polarises the travel of certain replicas (Figure 3 b), leading to a small divergence in the overall Δ*A* estimate of 0.11 kcal mol^−1^ (Table 2). Indeed, this also underlies the longer convergence required for **b3** through this re-weighting scheme (Figure 7 c).

Other free energy calculations with enhanced sampling MD methods have been applied in addressing the issue of pseudo-ergodicity and constitute promising alternatives; these include well-tempered metadynamics,^38^ replica exchange with solute tempering (REST2)^39^ and windowed aMD in a Hamiltonian replica exchange framework (w-REXAMD).^40^ Several biased MD approaches such as aMD use exponential re-weighting; for systems with large biasing energies, broad distributions of these energies lead to high energy terms with sizeable exponential weights that dominate the free energy estimates. Conversely, the weights of low energy terms are often lost in the limitations of numerical precision. There is indeed evidence of this energetic noise in the application of the stronger ses potential for **b3** here, which resulted in the broadest range in 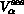
of the three systems. For applications where more dihedrals are enhanced by the ses potential, the larger biasing energy could potentially further increase the spread of 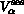
values and the accompanying uncertainty in free energy estimates. Clearly, further work is required to assess the optimal ses parameters and limiting system size for the recovery of accurate free energy profiles. In this regard, we note that the use of approximations to the exponential term such as cumulant expansion^41^ have shown utility in reducing noise in re-weighting,^42^ such that a narrow distribution is maintained, even for the enhancement of a greater number of degrees of freedom.

As a replica-based approach, sesTI affords a straightforward coupling of potentials, albeit with a judicious choice of parameters, and circumvents the energy overlap requirement of replica-exchange methods. In common with metadynamics, the sesTI approach requires a choice of coordinates to sample, in its current form a selection of dihedral angles. In principle, detailed knowledge of hidden, thermodynamically relevant conformations is not required a priori, but instead can be explored by using the swarm of coupled trajectories.

##  Conclusions

We have described a free energy simulation approach based on a swarm of coupled replicas to improve the underlying sampling of kinetically distinct states. Computational free energy changes for this transition using dual topology TI and IT-TI increasingly deviated from zero with increasing barrier height of intramolecular rotation. Alternatively, dual topology sesTI calculations applied enhanced sampling to the intramolecular dihedral of the reference and target states of butane, and led to computed free energy differences of zero for barrier heights up to 6 kcal mol^−1^. The sesMD simulations underlying these improved free energy change estimates displayed increased frequency of transitions between wells and greater coverage of phase space, as swarm replicas interacted to drive each other across energy barriers. The sesTI approach, therefore, shows potential in quantifying free energy differences in systems where the free energy change may depend on multiple distinct substates separated by non-trivial energy barriers.

## Supporting Information

As a service to our authors and readers, this journal provides supporting information supplied by the authors. Such materials are peer reviewed and may be re-organized for online delivery, but are not copy-edited or typeset. Technical support issues arising from supporting information (other than missing files) should be addressed to the authors.
