# Supplementary material for: Free Energy Calculations using a Swarm-Enhanced Sampling Molecular Dynamics Approach
Source: Chemphyschem. 2015 Sep 29;16(15):3233–41. doi: 10.1002/cphc.201500524 (PMC4676921; doi:10.1002/cphc.201500524)

CHEMPHYSCHEM

Supporting Information

Free Energy Calculations using a Swarm-Enhanced Sampling Molecular Dynamics Approach

Kepa K. Burusco,[a] Neil J. Bruce,[a, b] Irfan Alibay,[a] and Richard A. Bryce*[a]

cphc_201500524_sm_miscellaneous_information.pdf

Supporting Information

Table S1: GAFF parameters IDIVF, PK (kcal/mol), PHASE (deg) and PN, used in systems **b1**, **b2** and **b3**. The standard GAFF force field parameters are used for **b1**. In order to obtain **b2** and **b3**, the original value of PK was scaled by a factor of 1.6 and 2.2 respectively.

		c3-c3-c3-c3			hc-c3-c3-hc	hc-c3-c3-c3
		f1	f2	f3	f1	f1
**b1**	IDIVF	1.000	1.000	1.000	1.000	1.000
	PK	0.180	0.250	0.200	0.150	0.160
	PHASE	0.000	180.000	180.000	0.000	0.000
	PN	3.000	2.000	1.000	3.000	3.000
**b2**	IDIVF	1.000	1.000	1.000	1.000	1.000
	PK	0.288	0.400	0.320	0.240	0.256
	PHASE	0.000	180.000	180.000	0.000	0.000
	PN	3.000	2.000	1.000	3.000	3.000
**b3**	IDIVF	1.000	1.000	1.000	1.000	1.000
	PK	0.396	0.550	0.440	0.330	0.352
	PHASE	0.000	180.000	180.000	0.000	0.000
	PN	3.000	2.000	1.000	3.000	3.000

Figure S1: Rotational profile of butane potential energy for models **b1**, **b2** and **b3**

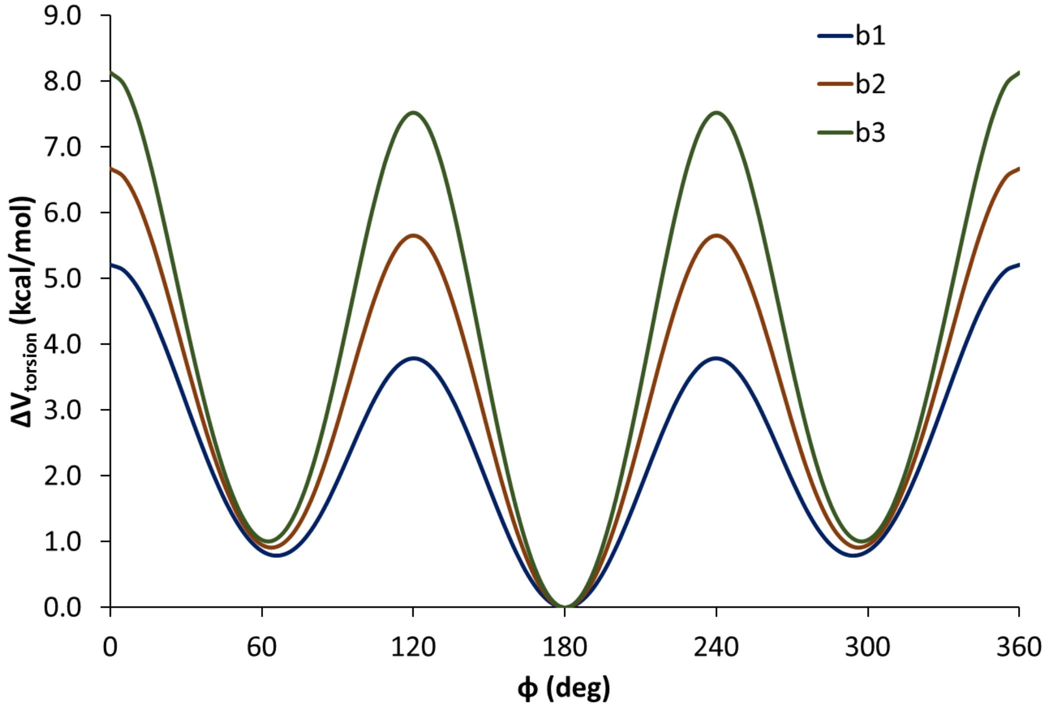

Supplement: Supplementary file 1 [file cphc0016-3233-sd1.pdf]
